# Prescribing or co-designing exercise in healthy adults? Effects on mental health and interoceptive awareness

**DOI:** 10.3389/fnbeh.2022.944193

**Published:** 2022-07-28

**Authors:** Maricarmen Almarcha, Ignacio González, Natàlia Balagué, Casimiro Javierre

**Affiliations:** ^1^Complex Systems in Sport Research Group, Institut Nacional d’Educació Física de Catalunya, University of Barcelona, Barcelona, Spain; ^2^Institut Nacional d’Educació Física de Catalunya, University of Barcelona, Barcelona, Spain; ^3^Departament de Ciències Fisiològiques, Facultat de Medicina i Ciències de la Salut, University of Barcelona, Barcelona, Spain

**Keywords:** personalized exercise, mental health, affective wellbeing, complex systems approach, network physiology of exercise, WHO recommendations, exercise adherence, self-awareness

## Abstract

Universal exercise recommendations for adults neglect individual preferences, changing constraints, and their potential impact on associated health benefits. A recent proposal suggests replacing the standardized World Health Organisation (WHO) exercise recommendations for healthy adults by co-designed interventions where individuals participate actively in the decisions about the selected physical activities and the effort regulation. This study contrasts the effects on mental health and interoceptive awareness of a co-designed and co-adapted exercise intervention with an exercise program based on the WHO recommendations for healthy adults. Twenty healthy adults (10 men and 10 women, 40–55 y.o.) participated voluntarily in the research. They were randomly assigned to a co-designed exercise intervention (CoD group) and a prescribed exercise program (WHO group). Supervised online by specialized personal trainers, both programs lasted 9 weeks and were equivalent in volume and intensity. The effects of the exercise intervention were tested through personal interviews, questionnaires (DASS-21 and MAIA) and a cardiorespiratory exercise test. Intragroup differences (pre-post) were assessed using the Mann-Whitney Wilcoxon test and intergroup differences through Student’s *t*-tests. Effect sizes were calculated through Cohen’s d. Interviews were analyzed through thematic analysis. Eleven participants completed the intervention (CoD = 8, WHO = 5). Both groups improved, but non significantly, their cardiorespiratory testing results, and no differences were found between them post-intervention. Mental health was only enhanced in the CoD group (*p* < 0.001), and interoceptive awareness improved in seven of the eight scales in the CoD group (*p* < 0.001) and only in 3 scales in the WHO group (*p* < 0.01). In conclusion, the co-designed intervention was more effective for developing mental health, interoceptive awareness, autonomy, and exercise self-regulation than the WHO-based exercise program.

## Introduction

Prescribing an adequate exercise program to promote health in the adult population has been a central research issue (Pollock et al., 2000). Two main types of programs (aerobic, resistance, and a combination of both) have been investigated due to their different physiological impact and proven benefits (Kenney et al., 2015). Based on the available research, the American College of Sports Medicine (ACSM) and the World Health Organization (WHO) have converged on the type of activity and minimum dose in their guidelines for exercise prescription in health and disease (Riebe et al., 2018) with light adaptations depending on the age and type of disease (Bull et al., 2020; Bushman, 2020). However, some authors have questioned the theoretical and methodological assumptions of this one-size-fits-all approach (Balagué et al., 2020a), and have proposed person-centered guidelines in line with current therapeutic tendencies (Carpinelli, 2009; Greenhalgh et al., 2014; Zimmer et al., 2018; Wackerhage and Schoenfeld, 2021).

The self-determination theory (SDT)-based exercise referral consultation (Jolly et al., 2009; Deci and Ryan, 2013) proposes interviewing participants before the exercise program about their perceptions of autonomy and motivational regulations. The aim is provide choices of activities, support initiations and relevant information for being physically active. Limiting their intervention to the beginning of the program, professionals do not guide adaptations during the program. However, a permanent interaction professionals-practitioners during this period may ensure adequate adjustments of exercise characteristics to keep participant’s wellbeing and adherence, and develop their self-knowledge and autonomy.

It is known that meaningful and motivating practices increase exercise adherence and, thus, its health-related effects (Salmon, 2001; Williams, 2008; Carek et al., 2011; Stonerock et al., 2015). Additionally, performing new and challenging activities seem to satisfy basic psychological needs (Fernández-Espínola et al., 2020).

The effects of regular exercise on depression and anxiety, the two most disabling mental disorders in both sexes, and among the top 25 leading causes of the global health-related burden (GBD 2019 Diseases and Injuries Collaborators, 2020) are well known. Mental health and emotional wellbeing improve through regular exercise (Paluska and Schwenk, 2000; Ströhle, 2009; Stanton and Reaburn, 2014; Schuch et al., 2016), and is also associated with a reduced risk of suffering from emotional disorders (Bernstein and McNally, 2018).

From a complex systems perspective, health has been defined as a dynamic product of the interplay between the external environment and internal physiology (Sturmberg et al., 2019). The health state can change in response to somatic conditions, social connectedness, emotional feelings, and subjective perceptions (Sturmberg, 2019). Within this paradigm, the stability of a healthy state can be achieved in multiple ways, and nonlinear, i.e., non-proportional, individual training effects may occur after exposure to exercise and training loads (Hristovski and Balagué, 2010). Even the same exercise intensity, which may promote positive adaptations in a specific person or context, may produce overtraining or no effects in another person or particular context (Hristovski et al., 2010).

The ACSM and the WHO recommend performing a minimum of 150 min of aerobic exercise at a moderate intensity or 75 min of vigorous-intensity exercise in combination with resistance training at least two times per week, using two sets of 8–15 repetitions with 60% of one-repetition maximum (American College of Sports Medicine, 2017; World Health Organization [WHO], 2020). These universal recommendations assume the existence of decontextualized realities (Jones et al., 2017) and ideal or prototypic fitness and health states. Although fitness is often associated with strength and conditioning, the fittest is not the strongest or endured in biological evolution but the most diverse (Pol et al., 2020). Accordingly, fitness has been redefined as the ability to survive in a broad range of contexts to adapt to socio-psycho-biological challenges (Balagué et al., 2020b; Hristovski and Balagué, 2020).

Even though most studies present favorable results applying the recommended types of exercise, systematic reviews and meta-analyses on exercise prescription indicate the lack of high-quality studies showing the sustainability of standardized programs (Sørensen et al., 2006) and the need for personalizing the recommendations (Zimmer et al., 2018). In line with personalized exercise medicine, it has been advised to reorient the main aims of exercise prescription and redefine the roles of health care exercise professionals interacting with users/patients (Balagué et al., 2020b). Notably, it has been suggested to develop the autonomy and self-regulation of users/patients through their active involvement in the co-design of exercise proposals and workload adjustments (Almarcha et al., 2021).

Due to substantial inter-individual differences and personal contexts, the development of interoceptive awareness of users/patients seems crucial for achieving self-knowledge and self-responsibility to select and regulate exercise workloads adequately (Pol et al., 2020; Montull et al., 2022). Interoceptive awareness is the ability to identify, access, understand, and respond appropriately to the patterns of internal signals that allow to engage in life challenges and ongoing adjustments (Craig, 2015). Regulating daily active and resting periods, frequency, intensity, and duration of exercise promotes healthy mind-body states. Almarcha et al. (2021) sustain that education on self-regulation of psycho-emotional and physical states is essential to promote health and affective wellbeing during home-based teleworking. The authors propose to healthcare professionals: (a) redefine fitness states, (b) refocus the aims of home-based exercise, (c) guide users from dependency to autonomy, (d) promote co-adaptive and co-learning processes, and (e) develop interoceptive awareness. As far as we know, no studies have evaluated the interoceptive awareness effects of a co-designed exercise program in healthy adults.

The present study aimed to contrast the effects of a co-designed exercise intervention with a WHO-based program on mental health and interoceptive awareness in healthy adults. Specifically, it was hypothesized that a co-designed intervention reduced further depression, anxiety, and stress levels and increased further interoceptive awareness and self-regulation.

## Materials and methods

### Participants

Twenty healthy adults (10 men and 10 women) aged between 40 and 50 y.o., with a medium socioeconomic status, higher education level and non-involved in regular physical activity, volunteered to participate in this study. Considering a target power of 0.8, a significance level of *p* < 0.05, and a medium effect size (0.5), the total sample size calculated using the software G * Power v3.1.9.7 was 12 participants. To compensate for the possible loss of follow-up, the proposed sample was 20 participants.

The exclusion criteria were the following: (a) report any contraindications and injury that prevented from physical exercise; (b) prohibitions of physical testing interventions; and (c) pacemaker dependent, not in sinus rhythm, pregnant, had a BMI greater than 30 kg/m^2^ or had known carotid or femoral artery stenosis.

After being informed about the study procedure and sign an informed consent, participants performed a cardiorespiratory exercise test and responded to two questionnaires (see below) through Google forms. All responses were coded to ensure data privacy and anonymity. After it, they were randomly divided into two groups of 10 members each that followed two different exercise programs during 9 weeks: (a) a co-designed exercise intervention (CoD group), and (b) a standardized exercise program based on WHO recommendations (WHO group). Both groups had similar fitness status and questionnaire scores. Participants were randomly assigned to 7 personal trainers (Sport Science graduates) with a minimum of 3 years of professional experience and specifically trained for 2 weeks to develop both intervention programs. Each coach trained virtually 2–3 participants. They supervised participants’ online training and interacted with them twice a week. They also had meetings with the research coordinator to follow up on the intervention weekly. The research project was approved by the Comité d’Ètica d’Investigacions Clíniques de l’Administració Esportiva de Catalunya (ref. 07/2015/CEICEGC). A total of 7 participants (CoD = 2, WHO = 5) did not complete the intervention program.

### Procedure

#### Co-designed exercise intervention

It had two main objectives: (a) promote health through co-designing meaningful and personally motivating activities, and (b) develop the participant’s autonomy, self-responsibility and interoceptive awareness. Participants proposed activities they liked to do or would like to try. Personal trainers also suggested activities that could be meaningful and motivating for the participant, not only those based on cyclic or repetitive movements (Almarcha et al., 2021). For example: playing with their kids, doing outdoor activities, playing exergames, stretching at the office, and using the bicycle for displacement, among others.

#### Standardized exercise program based on World Health Organization recommendations

It consisted of cycling or running exercises, performed 5 days per week at low intensity or 3 days per week at vigorous-intensity with a minimum duration of 25–30 min per session, according to the WHO guidelines (World Health Organization [WHO], 2019). The strength training consisted of a 12-station High-intensity circuit (Klika and Jordan, 2013) performed twice a week according to the American College of Sports Medicine Guidelines (ACSM) (Bushman, 2020). All exercises could be done with bodyweight in almost any setting (e.g., home, office, hotel room, etc.). Exercises were performed for 30 s, with 10 s of transition time between bouts. The total time for the entire circuit workout was approximately 7 min. The circuit could be repeated 2–3 times. Load intensity and volume were adjusted weekly to keep a rate of perceived exertion (Borg CR 10 Scale) of 3–5 in all sessions (see Table 1).

**TABLE 1:** Comparison of the characteristics of the exercise program based on WHO recommendations and the co-designed intervention.

Type of program	WHO exercise program (WHO)	Co-designed intervention (CoD)
**Foundation**	WHO and ACSM guidelines	Co-designed
**Exercise**	Repetitive	Varied
**Frequency**	Aerobic: 5 d/w	5 d/w
	Strength: 2 d/w	
**Intensity**	Aerobic: RPE (CR 10): 3–5	RPE (CR 10): 3–5
	Strength: RPE (CR 10): 8	
**Duration**	300 min/week	300 min/week
**Type of activity**	Aerobic (running, cycling), strength	Based on individual preferences

### Testing

A cardiorespiratory exercise test and two questionnaires (see below) were administered pre-and post-intervention, and a personal interview was performed at the end of the intervention. The cardiorespiratory exercise test was supervised by medical staff that confirmed the health status of the participants. The questionnaires were responded online using Google Forms, and an external researcher performed the personal video conference interviews.

#### Cardiorespiratory exercise test

An incremental cycling test (Excalibur, Lode, Groningen, Netherlands) starting at 50 W and increasing the workload by 30 W/min until reaching a subjective rate of perceived exertion (CR-10 scale) of “very hard” (RPE ≥ 8) was performed. Participants breathed through a valve (Hans Rudolph, 2700, Kansas City, MO, United States), and the respiratory gas exchange was determined using an automated open-circuit system (Metasys, Brainware, La Valette, France). Oxygen and CO2 content and airflow rate were recorded breath by breath. An electrocardiogram (ECG) was continuously monitored (DMS Systems, DMS-BTT wireless Bluetooth ECG transmitter and receiver, software DMS Version 4.0, Beijing, China). The test was performed in a well-ventilated lab; the room temperature was 23^°^C and the relative humidity 48%, with variations of no more than 1^°^C in temperature and 10% in relative humidity. The tests were carried out at least 3 h after a light meal, and participants were instructed not to perform vigorous physical activity for 72 h before testing.

#### Questionnaires

##### Depression, Anxiety and Stress scales (DASS-21)

The Spanish version of the DASS-21 questionnaire (Bados et al., 2005) assessed the self-reported negative emotional states during the last week. This questionnaire contains 21 statements rated on a four-categories of Likert scale (from 0 = “does not apply to me at all” up to 3 = “it applies a lot to me most of the time”), distributed along with three subscales (with seven items each): Depression, Anxiety and Stress (Lovibond and Lovibond, 1995). The depression scale assesses dysphoria, hopelessness, devaluation of life, lack of interest/involvement and inertia (e.g., I was unable to become enthusiastic about anything). The anxiety scale assesses autonomic arousal, skeletal muscle effects, situational anxiety, and subjective experience of anxious affect (e.g., I was aware of the action of my heart in the absence of physical exertion). The stress scale is sensitive to levels of chronic non-specific arousal. It assesses difficulty relaxing, nervous arousal, and being easily upset/agitated, irritable/over-reactive and impatient (e.g., I was intolerant of anything that kept me from getting on with what I was doing).

Scores for depression, anxiety and stress are calculated by summing the scores for the relevant items. The values of Cronbach’s alpha and test-retest reliability indicate good internal consistency of the Spanish version of DASS-21 (Bados et al., 2005).

##### Multidimensional Assessment of Interoceptive Awareness

The Spanish version of the multidimensional assessment of interoceptive awareness (MAIA) questionnaire (Valenzuela-Moguillansky and Reyes-Reyes, 2015) was administered. It is a self-report instrument developed by Mehling et al. (2012) to measure eight dimensions of interoceptive body awareness. The noticing scale assesses the awareness of uncomfortable, comfortable or neural body sensations (e.g., when I am tense, I notice where the tension is located in my body). The not distracting scale assesses the tendency not to use distraction to cope with discomfort (e.g., when I feel pain or discomfort, I try to power through it). The not worrying scale assesses the tendency to not experience emotional distress with physical discomfort (e.g., I can notice an unpleasant body sensation without worrying about it).

The attention regulation scale assesses the ability to sustain and control attention to body sensations, (e.g., I can maintain awareness of my inner bodily sensations even when a lot is going around me). The emotional awareness scale assesses the ability to attribute specific physical feelings to physiological manifestations of emotions (e.g., When something is wrong in my life, I can feel it in my body). The self-regulation scale assesses the ability to regulate distress by attention to body sensations (e.g., I can use my breath to reduce tension). The body listening scale assesses the tendency to actively listen to the body for insight (e.g., I listen to my body to inform me about what to do). The trusting scale assesses the experience of one’s body as safe and trustworthy (e.g., I feel my body is a safe place). It has 32 items tested on a Likert scale, with six levels of ordinal response coded from 0 (never) to 5 (always), generating a total direct score on a scale that ranges from 0 to 160 points. The Spanish version showed appropriate construct validity and reliability indicators, with a Cronbach’s α of 0.90 for the total scale and values between 0.40 and 0.86 for the different subscales (Valenzuela-Moguillansky and Reyes-Reyes, 2015).

#### Interviews

Participants were invited to a 30-min semi-structured video conference interview by a researcher external to the intervention to avoid bias. The semi-structured interview guides the intervention’s critical points while allowing new themes/issues to emerge. The reliability of the interviews was guaranteed by the same interviewer, the same design scheme of the questions, the same length of interrogation and the same period for all interviews. The interviews were video recorded and transcribed verbatim. Two experienced qualitative researchers and five Sport Science professionals validated the interview guide before the study. It consisted of questions concerning: previous experiences, reasons for involvement in the exercise program, perceived benefits or challenges to participating, and the role of the personal trainer (e.g., Do you think the figure of the personal trainer is essential to continue with the level of physical activity achieved?) reasons to drop out/adherence, what they have learned (e.g., Have you gained resources to adapt the exercise to any personal and environmental situation?) and readiness to implement changes in their lifestyle behavior beyond the program (e.g., Have you discovered any physical activities to incorporate into your habits?).

### Data analysis

#### Physiological testing

The ratio between the workload corresponding to the RPE ≥ 8 and the oxygen uptake relative to body weight (VO_2_) at this workload (W/VO_2_) and the ratio between the workload corresponding to the RPE ≥ 8 and the heart rate (HR) at this workload (W/HR) were compared pre-and post-intervention using Wilcoxon matched-pairs test and independent samples *t*-test.

#### Questionnaires

Means with 95% confidence intervals, medians, standard deviations, and interquartile ranges were calculated to assess the location and dispersion of scores obtained in DASS-21 and MAIA. Skewness and kurtosis were used to determine the shape and normality of the distribution of scores. Intragroup pre-post differences were evaluated using paired *t*-tests and intergroup differences through independent samples *t*-tests. Effect sizes were calculated through Cohen’s d. All quantitative statistical analyses were conducted using SPSS (version 25, IBM Corp).

#### Interviews

All interview transcripts were cross-checked with video recordings to ensure accuracy. Identification codes were assigned [WHO, CoD or NC -non-completer-, with consecutive numbers (e.g., CoD4)]. Thematic analysis was employed to identify emergent themes until saturation was reached (Braun and Clarke, 2006). Two researchers independently reviewed one transcript to identify codes using NVIVO software (version 2, QRS International Pty Ltd). Then, they discussed the findings and presented the proposed principles derived from the data to the research team until consensus was reached on the final regulations.

## Results

### Physiological testing

No differences in W/VO_2_ and W/HR were found pre-post intervention in both groups (WHO and CoD) nor between groups.

### Questionnaires

All participants responded to DASS-21 and MAIA questionnaires, but only 13 of them completed the intervention (CoD = 8, WHO = 5).

#### Depression, anxiety and stress levels

DASS questionnaire descriptives are presented in Table 2. After the intervention, the CoD group improved their scores in the three dimensions of the DASS-21 questionnaire: depression [*t*(7) = 7.907, *p* = 0.0001], anxiety (*t* = 27.032, *p* = 0.0001) and stress (*t* = 8.973, *p* = 0.0001). However, no differences were detected in the WHO group. Intergroup differences showed higher anxiety (W = 36.000, Z = –3.038, *p* = 0.002, d = 0.84) and stress (W = 39.500, Z = –2.449, *p* = 0.011, d = 0.68) in the WHO group compared to the CoD group.

**TABLE 2:** DASS questionnaire items descriptives.

	Group	*N*	Mean	*SD*	Variance	Skewness	SE	Kurtosis	SE
PRE_Depression	CoD	8	12.250	4.46	19.93	1.0261	0.752	–0.234	1.48
	WHO	5	8.400	7.80	60.80	1.9861	0.913	3.948	2.00
POST_Depression	CoD	8	1.500	1.41	2.00	0.4041	0.752	–0.229	1.48
	WHO	5	6.000	4.24	18.00	–0.5238	0.913	–0.963	2.00
PRE_Anxiety	CoD	8	13.000	1.51	2.29	–1.3229	0.752	0.875	1.48
	WHO	5	9.200	5.93	35.20	–0.8849	0.913	1.449	2.00
POST_Anxiety	CoD	8	0.750	1.04	1.07	0.6441	0.752	–2.240	1.48
	WHO	5	10.000	2.45	6.00	1.3608	0.913	2.000	2.00
PRE_Stress	CoD	8	17.750	4.83	23.36	–0.5353	0.752	–0.744	1.48
	WHO	5	8.800	5.76	33.20	–0.0376	0.913	–1.804	2.00
POST_Stress	CoD	8	4.000	3.02	9.14	0.3307	0.752	–1.488	1.48
	WHO	5	14.400	9.42	88.80	1.4646	0.913	2.443	2.00

#### Interoceptive awareness

MAIA questionnaire descriptives are presented in Table 3. After the intervention, the CoD group improved in 7 of the 8 scales of the MAIA questionnaire: noticing [*t*(7) = –1.276, *p* = 0.0001], not-distracting [*t*(7) = –4.492, *p* = 0.003], attention regulation [*t*(7) = –26.839, *p* = 0.0001], emotional awareness [*t*(7) = –13.642, *p* = 0.0001], self-regulation [*t*(7) = –13.316, *p* = 0.0001], body listening [*t*(7) = –7.848, *p* = 0.0001], trusting [*t*(7) = –10.991, *p* = 0.0001] except for not-worrying [*t*(7) = –1.276, *p* = 0.243]. In the WHO group, differences were detected only in 3 scales, self-regulation [*t*(4) = 4.376, *p* = 0.012], body listening [*t*(4) = –6,144, *p* = 0.004], and trusting [*t*(4) = –6,364, *p* = 0.003].

**TABLE 3:** MAIA questionnaire items descriptives.

	Group	*N*	Mean	*SD*	Variance	Skewness	SE	Kurtosis	SE
PRE_Noticing	CoD	8	1.91	0.7063	0.4989	–0.7578	0.752	2.4997	1.48
	WHO	5	2.10	0.5477	0.3000	1.5310	0.913	1.7448	2.00
POST_Noticing	CoD	8	4.25	0.7440	0.5536	–1.2140	0.752	1.5595	1.48
	WHO	5	4.55	0.4472	0.2000	–0.0524	0.913	–2.3242	2.00
PRE_Not- distracting	CoD	8	2.92	0.3897	0.1518	1.3533	0.752	0.6000	1.48
	WHO	5	2.80	0.1807	0.0327	0.6086	0.913	–3.3333	2.00
POST_Not-distracting	CoD	8	4.08	0.6357	0.4041	–1.7759	0.752	4.0011	1.48
	WHO	5	4.27	0.4349	0.1891	–0.5215	0.913	–1.5244	2.00
PRE_ Not-worrying	CoD	8	2.08	1.3060	1.7056	0.3178	0.752	–0.1760	1.48
	WHO	5	2.20	1.3431	1.8039	1.0194	0.913	2.0429	2.00
POST_Not-worrying	CoD	8	2.59	0.3908	0.1527	–0.8853	0.752	–0.4606	1.48
	WHO	5	2.73	0.0158	2.50e-4	1.40e-14	0.913	–1.2000	2.00
PRE_ Attention regulation	CoD	8	2.10	0.5165	0.2667	0.4576	0.752	–0.4061	1.48
	WHO	5	2.08	0.6106	0.3728	–1.0217	0.913	0.6871	2.00
POST_ Attention regulation	CoD	8	4.08	0.5560	0.3091	–0.3548	0.752	–0.6512	1.48
	WHO	5	3.37	0.9125	0.8326	0.1683	0.913	–1.1433	2.00
PRE_Emotional awareness	CoD	8	2.50	0.6928	0.4800	0.2887	0.752	–0.9250	1.48
	WHO	5	3.36	0.6542	0.4280	–2.1342	0.913	4.6790	2.00
POST_ Emotional awareness	CoD	8	4.42	0.5701	0.3250	–0.3154	0.752	–1.7276	1.48
	WHO	5	4.00	1.1489	1.3200	–1.0550	0.913	0.9669	2.00
PRE_Self-regulation	CoD	8	2.16	0.6400	0.4096	0.2161	0.752	–0.1049	1.48
	WHO	5	2.60	1.3062	1.7063	0.5861	0.913	–0.3385	2.00
POST_ Self-regulation	CoD	8	4.38	0.6124	0.3750	–0.7776	0.752	–0.0571	1.48
	WHO	5	3.70	1.0216	1.0437	–0.3473	0.913	0.4149	2.00
PRE_Body-Listening	CoD	8	2.04	1.3499	1.8222	–0.0175	0.752	–0.9724	1.48
	WHO	5	1.73	0.9269	0.8591	0.2371	0.913	–0.8817	2.00
POST_ Body-Listening	CoD	8	4.12	0.6657	0.4431	–0.1099	0.752	0.0280	1.48
	WHO	5	3.67	1.1058	1.2228	–0.6821	0.913	1.1194	2.00
PRE_Trusting	CoD	8	2.58	0.6117	0.3741	0.6851	0.752	–0.4693	1.48
	WHO	5	2.73	1.3650	1.8631	1.5223	0.913	2.5324	2.00
POST_Trusting	CoD	8	4.42	0.4961	0.2461	0.4799	0.752	–2.2481	1.48
	WHO	5	4.00	1.0277	1.0561	–1.2840	0.913	2.0199	2.00

Post intervention inter-groups differences were observed in noticing (W = 20.500, Z = –2.140, *p* = 0.030, d = 0.59), non-distracting (W = 18.500, Z = –2.442, *p* = 0.011, d = 0.68), attention regulation (W = 16.000, Z = –2.789, *p* = 0.003, d = 0.77), self-regulation (W = 15.000, Z = –2.956, *p* = 0.002, d = 0.81), listen to body signals (W = 15.000, Z = –2.952, *p* = 0.002, d = 0.82) and trust (W = 15.000, Z = –3.029, *p* = 0.002, d = 0.84) in favor of the CoD group.

### Interviews

**FIGURE 1:**
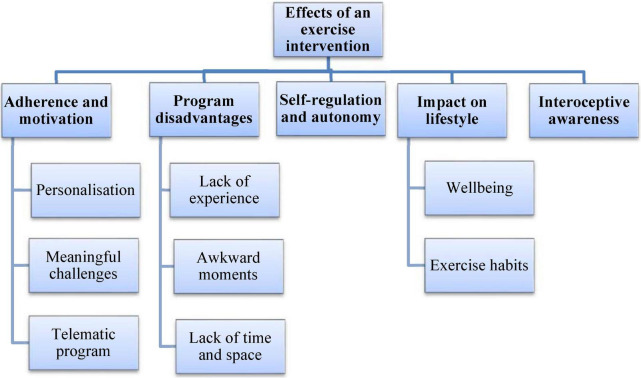
Themes and subthemes developed from the experiences of people participating in the exercise programs.

All participants were interviewed, including those who did not complete the entire intervention. The thematic analysis confirmed data saturation at *n* = 20 and revealed five themes based on the interview questions (see Figure 1).

### Theme 1: Adherence and motivations during the program

Both groups concurred that being supervised by the personal trainer and receiving constant feedback facilitated their participation. Highlighting the personalization of the co-design was felt very motivating and helped to continue. They also appreciated the meaningful and novel challenges; “Not knowing what challenges the personal trainer was going to present to me next week kept me curious and motivated” (CoD8). A telematic exercise program was another widely reported benefit “”It was not necessary to go to a specific place; I could do the exercise at home during my free time” (WHO2).

### Theme 2: Disadvantages of the program

Participants of the WHO group reported anxiety and stress due to their lack of experience exercising. “At the beginning, it was stressful because I am not used to it, and I did not feel fit” (NC5). As they principally exercised at home or work, they felt embarrassed when being observed by members of their families or co-workers; “I went to the bathroom to do some exercises so that my colleagues in the office could not see me” (CoD6). “During the Christmas holidays, I had my family at home, and I cared about what they could say when seeing me exercising” (NC3). The work schedule made participation difficult, and lack of time was the most reported problem in both groups; “I have little free time and find it difficult to combine exercise with family life. Physical activity is not a priority for me” (WHO4). Non-completers reported a lack of space or time to exercise; “Initially I felt very excited, but afterward it was difficult to get along and enjoy it due to my work schedule” (NC4). They dropped out of the study due to the program’s lack of schedule flexibility and incompatibility; “It has been impossible for me to combine it with work and family issues” (NC6). Most participants of the CoD group, which also encountered the time barrier, succeeded in adapting to it by incorporating physical activity into their daily life in an autonomous way. “On the first day, I walked only for 30 min, but as the weeks went by, I increased the time as I felt better every day” (CoD6).

### Theme 3: Self-regulation and autonomy

Participants of the CoD group adapted the exercise to their personal and environmental constraints and learnt how to self-regulate workload. “During holidays, I was not used to exercising, but this time I was able to do it because I knew the activities I could do” (CoD2) and “Being able to self-manage the activity’s intensity and duration helped me do much more than I thought at first” (CoD2). They did not perceive the figure of the coach as essential due to the learnt autonomy “As time passed I was able to decide daily which activities were suitable and which not” (CoD1). The WHO group did not report self-regulation and autonomy capacities. “Although I was stressed by work, I did the series and repetitions of the strength workout I had to do” (WHO5). Non-completers found it challenging to decide on the exercise program and considered they were assuming too much responsibility; “It is impossible for me to self-regulate the intensity and the type of activity because I am always so tired that I would never do anything” (NC2).

### Theme 4: Impact on lifestyle

The CoD group increased self-awareness and wellbeing: “I notice that when I walk, I get less tired, and I also notice fewer arrhythmias from the program’s start” (CoD6). Many discussed their plans for exercising due to their wellbeing and trust “I feel good about myself and my body” (CoD5). Also, some of them have hired a personal trainer to continue exercising; “I have decided to continue with a personal trainer to try new different activities that I have always wanted to try” (CoD7). Other participants expressed having gained body awareness “I am now more aware of the physical activity I do in my daily life, and how I feel during and after doing it” (CoD2). “I am still exercising, incorporating my trainer’s principles” (CoD3).

The WHO group also planned to change their habits by joining a gym where finding the required material. “I have decided to join a gym to access more material; it is easier for me to follow exercise routines” (WHO5). In line with the results of the questionnaires, “I have found that exercise helps reduce my anxiety and stress levels” (WHO3).

### Theme 5: Interoceptive awareness

While the WHO group linked the intervention to their physical fitness, the CoD group linked it to multiple dimensions and a more general wellbeing state: “For me, health is more than a physical state. It is about being comfortable with yourself and knowing how to listen to your body to adapt to different contexts” (CoD7). Both groups stated that they had never been asked about their body state and sensations towards exercise. However, only the CoD group mentioned they had improved their interoceptive awareness; “I can recognize inflammatory states in my body based on stress, what I have eaten or what I have slept” (CoD5). “When I am nervous, I cannot trust my body signals” (WHO2).

Overall, 13 out of 20 participants were satisfied with the exercise program. However, only participants of the CoD group would recommend the program to other people; “Thanks to the constant dialogue with the personal trainer and trying different proposals, I have discovered the types of activities that work for me. Actually, I have incorporated it as a daily habit” (CoD3); while others would have preferred daily monitoring and comprehensive training planning; “I prefer to have a programmed exercise routine with technique instructions” (CoD7).

## Discussion and conclusion

This study contrasts the effects on mental health and interoceptive awareness of co-designed exercise interventions and prescribed exercise programs based on the WHO recommendations for healthy adults. The co-designed intervention, aiming to guide participants from dependency to autonomy, promoted further mental health and interoceptive awareness, crucial aspects of cognitive and emotional wellbeing.

In alignment with previous research, the current study supports that exercise benefits mental health in adult population (Carek et al., 2011; Rebar et al., 2015; Bernstein and McNally, 2018). However, in this study, only participants of the CoD group reduced depression, anxiety and stress levels after the intervention. The duty to follow the program (i.e., “to take the exercise pill”), trying to comply strictly with WHO guidelines and following the exact doses contributed to increased anxiety and stress levels in some participants of the WHO group, These adverse effects have not been observed in participants performing new and challenging activities (González-Cutre et al., 2019).

In addition, only the CoD group expressed the wish to continue the intervention in the future. This can be explained by the positive affective valence reported during and after exercise by this group (Ekkekakis et al., 2011). In contrast, the emotional experience of the WHO group was not helping to introduce changes in their daily routine, as found in previous works (Kwan and Bryan, 2010; Antoniewicz and Brand, 2016). The pressure to follow a fixed exercise program, independently of daily constraints (e.g., holiday periods, family visits, amount of work), and the lack of novelty in the exercise routine may explain such results (Cao et al., 2021). As confirmed through the interviews, the five WHO participants who dropped out of the intervention reported getting overwhelmed when they could not comply with the prescribed routine and feel bored for doing the same type of exercise. Monotony seems a crucial factor contributing to low physical activity participation (Dalle Grave et al., 2011). Even active individuals engaged in the same exercise over time can reduce their motivation to the practice because of boredom (Lakicevic et al., 2020).

Enjoyment and interest seem determinant for ensuring long-term engagement (Argent et al., 2018) and adherence to exercise, leading ultimately to better health (Jekauc, 2015; Lakicevic et al., 2020). For example, performing similar physical activities in groups or virtual reality environments (e.g., exergaming approach) can create a distraction from negative thoughts related to exercise and encourage participation (Molina et al., 2014). Other authors propose an agreement between the athlete-trainer on goals and the selection of exercises to fit the athlete’s needs, motivations, and abilities (Wackerhage and Schoenfeld, 2021). Some results have shown more outstanding fitness scores and affective responses when participants self-selected exercise conditions (Parfitt et al., 2006) or when designing their exercise program based on their previous knowledge instead of following a standardized exercise program (Papadakis et al., 2021).

Decisions to engage in regular physical activity are also influenced by participants’ expectancies (Loehr et al., 2014). It is worth pointing out that the two CoD participants dropping out during the intervention expected to get involved in a standardized training program to become stronger. The literature refers to previous interventions, based on the SDT (Deci and Ryan, 2013), for increasing the motivation and adherence of patients to exercise treatments. Positive effects on adaptive motivational processes, quality of life and wellbeing have been reported (Duda et al., 2014). In fact, when participants self-paced or chose their mode of exercise, have more positive affective responses and autonomy than when they follow exercise prescriptions (Parfitt and Gledhill, 2004).

Although both (SDT practices and CoD intervention) consider participants’ motivations and preferences, the CoD goes a bit further, proposing a change of roles: participants and professionals co-design and co-adapt continuously unique and personalized protocols to develop participants’ self-knowledge and autonomy. That is, the initial program may evolve during the intervention due to the need of co-adapting to the multiple personal and environmental constraints affecting the process. For instance, a participant evolved from proposing home-based exercises to open-air activities in family and activities in nature (e.g., climbing mountains, trekking, etc.).

As confirmed through the interviews, the role of exercise co-designers developed in the CoD group self-knowledge, self-regulation, and autonomy. These potentially beneficial learning effects can also be associated with rewarding experiences (Fisher and Yarwood, 2008; Leckey, 2011). Being less dependent on the personal trainer, facilities and equipment, they could potentially create a larger adaptivity and guarantee a longer adherence to exercise. As they could choose the physical and social environments for exercising (e.g., in a park, in the mountains, with family, or friends), they could explore different exercise modalities, experience the most contextually adequate, and become more aware of their wellbeing and health.

It is worth pointing out that the commonly prescribed exercise for the adult population, converging on aerobic and strength programs and adhering to WHO recommendations, is based on oversimplified theoretical and methodological assumptions, as the possibility to transfer to individuals results obtained by comparing group data means (Balagué et al., 2020b). The network physiology of exercise conceptualizes the human organism as a complex adaptive system embedded and in constant interaction with social and policy factors (Bauman et al., 2012). Assuming there is no unique way to promote a healthy state (Sturmberg et al., 2019), some authors propose co-designing contextually sensitive, meaningful, motivating, and self-regulated exercise programs (Balagué et al., 2020a). However, as far as we know, few attempts have been made to contrast the emotional benefits of different exercise interventions (Hogan et al., 2015).

Concerning the interoceptive awareness effects, the light results obtained in the MAIA questionnaire by the WHO group (improving only in three of the eight scales), compared to the CoD group (seven of eight), can be explained by the lack of emphasis on the WHO intervention on interoceptive awareness and the aforementioned stressful experiences of participants. Stressed individuals show difficulties regulating emotional responses because they stopped trusting and listening to body signs (Price and Hooven, 2018). In contrast, the CoD group improved in seven of the eight scales and had fewer difficulties adapting the exercise type and characteristics to their personal and environmental constraints. An exciting outcome of performing novelty, motivating and challenging activities is the deep level of engagement which can lead to flow experiences (Swann et al., 2019; Montull et al., 2020), which increase autonomous motivation, reduces boredom, and relates to future involvement (Csikszentmihalyi, 2020).

Recent sports-related research has highlighted the need to develop early education programs to enhance interoceptive awareness (Montull et al., 2022). Our results show that a co-designed type of intervention may help to develop this relevant property. Unfortunately, no studies have tested the potential benefits of interoceptive awareness and self-knowledge on fitness states. We hypothesize a circular causality between both variables that need to be explored.

This research has some potential practical implications for future exercise interventions in adult population: (a) meaningful, motivating, and enjoyable activities may produce more psychological benefits than universal exercise recommendations, and lead to further exercise adherence, (b) initial exercise programs should be co-adapted to the changing personal and environmental constraints, not the other way around, (c) practitioners perceptions and interoceptive awareness should be developed to help on selecting adequately the type and characteristics of exercise and be the basis for workload adaptation. In turn, health professionals and personal coaches should: (a) be aware of the limitations of current exercise guidelines and be educated on co-designing instead of prescribing exercise, (b) lead practitioners from dependency to autonomy providing training criteria instead of exercise receipts, (c) contribute to the development of interoceptive awareness to prevent injuries, detect deleterious training effects (e.g., like overtraining) and promote a multidimensional fitness state (Almarcha et al., 2021).

It is recommendable to investigate the effects of co-designed exercise interventions on other populations like chronic patients and different age groups. In addition, a gender analysis, which was impossible to implement in this research due to the small number of participants, is also warranted.

It was expected to find more noticeable physiological effects of the intervention in the WHO group because participants were tested through cycle ergometers. While cycling was recommended in the aerobic training routines of the WHO group, the CoD group did not specifically practice cycling. Due to the low sample, no significant differences were found in cycling performance and physiological associates either inter or intra-groups. Further research is warranted to study the long-term effects of this type of intervention.

In conclusion, a co-designed exercise intervention was more effective for developing mental health, interoceptive awareness, autonomy, and exercise self-regulation in healthy adults than the prescription of an exercise program based on the universal recommendations of the WHO.

## Data availability statement

Publicly available datasets were analyzed in this study. This data can be found here: doi: 10.6084/m9.figshare.19767163.v1.

## Ethics statement

The studies involving human participants were reviewed and approved by Comité d’Ètica d’Investigacions Clíniques de l’Administració Esportiva de Catalunya (ref. 07/2015/CEICEGC). The patients/participants provided their written informed consent to participate in this study.

## Author contributions

MA, NB, and CJ contributed to the conception and design of the study. MA and IG organized the database and wrote the first draft of the manuscript. MA performed the statistical analysis. MA and NB wrote the final manuscript. All authors contributed to manuscript revision, read, and approved the submitted version.
